# Duration and outcome of orthotic treatment in children with clubfoot – a four-year follow-up national register study of Swedish children born between 2015 and 2017

**DOI:** 10.1186/s12891-024-07544-5

**Published:** 2024-05-31

**Authors:** Josefine Eriksson Naili, Malin Lindeberg, Anna-Clara Esbjörnsson

**Affiliations:** 1 Department of Women’s and Children’s Health, Karolinska Institutet, Stockholm, Sweden; 2 Motion Analysis Lab, Karolinska University Hospital, Stockholm, Sweden; 3 Department of Orthopedics, Skane University Hospital, Lund, Sweden; 4 Department of Clinical Sciences and Infectious Diseases, Skane University Hospital, Lund University, Lund, Sweden

**Keywords:** Congenital talipes equinovarus, Pes equinovarus adductus, Orthoses, Outcome, Foot abduction brace, Epidemiology

## Abstract

**Background:**

The Ponseti method for treating clubfoot consists of initial treatment with serial casting accompanied by achillotenotomy if needed, followed by the maintenance phase including treatment with a foot abduction orthosis (FAO) for at least four years. This study aimed to examine the duration, course, and outcome of orthotic treatment in children with clubfoot.

**Methods:**

321 children with clubfoot, born between 2015 and 2017, registered in the Swedish Pediatric Orthopedic Quality Register (SPOQ), were included in this prospective cohort study. Data on deformity characteristics and orthotic treatment were extracted. For children with bilateral clubfoot, one foot was included in the analysis.

**Results:**

Of the 288 children with isolated clubfoot, 274 children (95.5%) were prescribed an FAO, and 100 children (35%) changed orthosis type before 4 years of age. Of the 33 children with non-isolated clubfoot, 25 children (76%) were prescribed an FAO, and 21 children (64%) changed orthosis type before 4 years of age. 220 children with isolated clubfoot (76%), and 28 children with non-isolated clubfoot (84%) continued orthotic treatment until 4 years of age or longer. Among children with isolated clubfoot, children ending orthotic treatment before 4 years of age (*n* = 63) had lower Pirani scores at birth compared to children ending orthotic treatment at/after 4 years of age (*n* = 219) (*p* = 0.01). It was more common to change orthosis type among children ending orthotic treatment before 4 years of age (*p* = 0.031).

**Conclusions:**

The majority of children with clubfoot in Sweden are treated with an FAO during the maintenance phase. The proportion of children changing orthosis type was significantly greater and the Pirani score at diagnosis was lower significantly among children ending orthotic treatment before 4 years of age. Long-term follow-up studies are warranted to fully understand how to optimize, and individualize, orthotic treatment with respect to foot involvement and severity of deformity.

**Level of evidence:**

II.

**Supplementary Information:**

The online version contains supplementary material available at 10.1186/s12891-024-07544-5.

## Introduction

Clubfoot is a common congenital orthopedic pediatric foot deformity [1], characterized by equinus of the ankle, varus of the hind foot, cavus, and forefoot adduction with associated atrophy of the calf muscles [2]. Clubfoot commonly presents as isolated congenital, even though the condition may be associated with other conditions such as myelomeningocele or arthrogryposis [3]. The cause is considered to be multifactorial, including both genetic and environmental factors [3]. In Sweden, around 1.35 children/1,000 live births are born with clubfoot, including both isolated and non-isolated cases. Of the children with isolated clubfoot, around 74% are boys and 47% have bilateral involvement [4]. Between the years 2016 and 2019, 8% of children born with clubfoot in Sweden were reported to have clubfoot in combination with other diseases, referred to in this article as non-isolated [4].

The Ponseti method is currently considered the gold standard treatment of both isolated and non-isolated clubfoot [5]. Initial treatment includes weekly stretches, manipulations, and casting, accompanied by achillotenotomy if needed [6]. According to the Ponseti method, five to seven casts are usually needed to correct a clubfoot in a child with an isolated clubfoot [7]. For children with non-isolated clubfoot or clubfeet with atypical signs, a modified casting technique and sometimes additional number of casts are required [8, 9]. Initial treatment typically starts within the first weeks of life, and is followed by orthotic treatment for four to five years [10], which is termed the maintenance phase, and is the primary focus of this study.

The Swedish Pediatric Orthopedic Quality Register (SPOQ), founded in 2015, covers five common pediatric diagnoses of which clubfoot is one [11]. This national prospective total cohort register aims to obtain generalizable knowledge, and to improve treatment and outcomes for all children with the included diagnoses. In SPOQ clubfoot section, foot deformity before and after treatment, and details of initial treatment, e.g. type of orthosis and prescribed time of orthotic use, are reported according to a standardized protocol. The gold standard treatment for children with clubfoot born in Sweden is, and was between 2015 and 2017, the Ponseti Method [12]. The Ponseti method has improved clubfoot treatment, with a described drop in need for surgical interventions, during and following initial treatment and maintenance phase [8, 13, 14]. Despite successful treatment, recurrence of the initial foot deformity is common, particularly among those non-compliant to orthotic treatment [15–22]. Therefore, a systematic analysis of the treatment provided, and how the recommended treatment is being followed may provide insights to further improve orthotic treatment in children with clubfoot.

The primary aim of this study was to examine the duration, course, and outcome of orthotic treatment in children with isolated and non-isolated clubfoot deformity in Sweden using a national cohort of children born between 2015 and 2017. Secondly, we aimed to analyze change of orthosis type and end of orthotic treatment with respect to severity of clubfoot deformity at diagnosis and uni- or bilateral involvement in children with isolated clubfoot.

### Ethics statement

This study was approved by the Swedish Ethical Review Authority, DNR: 2019–04989. Details applying to this ethical approval are reported elsewhere [4]. Unidentified data was accessed the 1st of September 2022, and participants could not be identified by the authors.

## Materials and methods

At birth, upon suspicion of clubfoot, the child is referred to one of the 28 pediatric orthopedic centers treating clubfoot in Sweden. In 2017, 27 of these centers register data in SPOQ, between the years 2015 to 2017 the coverage increased from 40 to 96% [11]. Since almost all children in Sweden are born at a hospital, there are practically no undiagnosed cases. To validate the number of children with clubfoot registered in SPOQ, these numbers were compared with those in the Swedish national patient register and the average national completeness was 82% in 2017 [11].

### Participants

After a clubfoot diagnosis, children with isolated or non-isolated clubfoot are registered in SPOQ by the treating hospital. The inclusion and exclusion criteria for entry in SPOQ are described in detail elsewhere [4]. Between the 1st of January 2015 and the 31st of December 2017, 387 children with clubfoot (a total number of 579 clubfeet) were registered in SPOQ. All children with clubfoot registered in SPOQ at birth and at 4 years of age were included in the present study.

### Studied parameters

The following parameters were extracted from SPOQ: Number of children with isolated clubfoot and non-isolated clubfoot; Gender; Uni- or bilateral involvement; Presence of atypical signs before the start of treatment (described in detail elsewhere [4]); Pirani score at diagnosis; Type of prescribed orthosis; Time of prescribed orthotic use; Parent-reported hours of orthotic use; Change of orthosis type; End of orthotic treatment; and Pirani Böhm Sinclair (PBS)-score at 4 years of age.

### Non-isolated clubfoot

In SPOQ, clubfeet associated with other diseases (non-isolated clubfoot) are reported upon entry in the register in one of the following categories: Arthrogryposis multiplex congenital Q74.3; Spina bifida Q05.9; Congenital malformation syndromes predominantly involving limbs Q87.2; Neurological diseases (not specified); and Other (not specified). The number of clubfeet associated with other diseases were adjusted based on updated reports at the one-year follow-up.

### The Pirani score

Clubfoot deformity is often classified at the first visit to specialized health care and after casting treatment according to the Pirani score. The Pirani score is a disease-specific foot deformity classification system, scoring the foot deformity from 0 (no foot deformity) to 6 (maximum foot deformity) [23–25]. Feet with a score of less than 1 are classified as positional clubfeet or other minor foot deformities, and these are not possible to register in SPOQ [11].

### Orthotic treatment

**Fig. 1:**
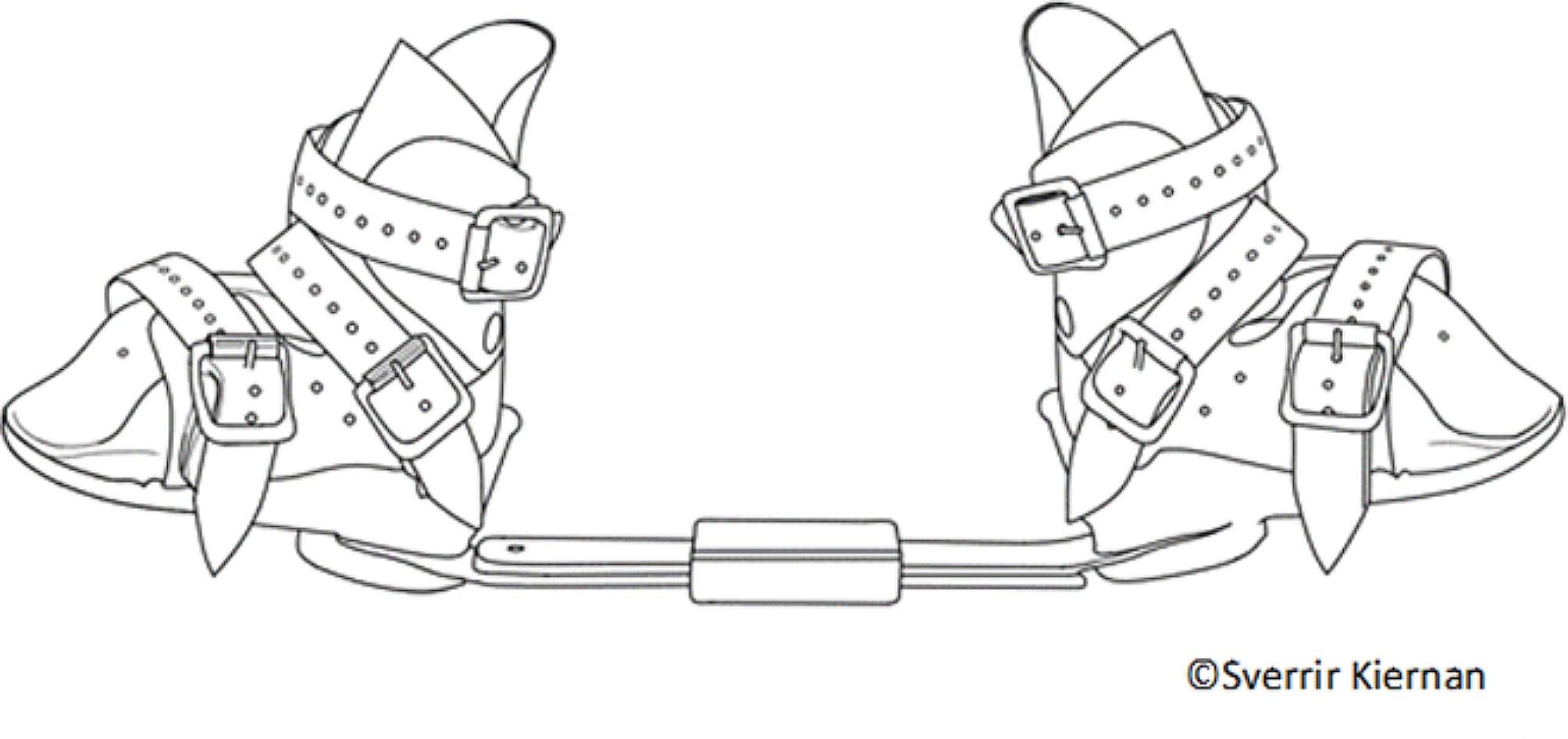
A Foot Abduction Orthosis (FAO), the first choice according to the Ponseti Method. The FAO consists of two shoes, outward rotated to 60 degrees, connected by a bar. © Sverrir Kiernan

In this study, orthotic treatment was defined as the treatment period following directly after the cast treatment for at least four consecutive years (termed the maintenance phase). According to the Ponseti method, the initial treatment is followed by treatment with a foot abduction orthosis (FAO) as the first choice (Fig. 1). Other types of orthoses that may be utilized if the FAO is not accepted by the child, or for other reasons, include knee-ankle-foot-orthosis (KAFO) and ankle-foot-orthosis (AFO).

### Parent-reported hours of orthotic use and change of orthosis type

During a clinical follow-up, when the child was aged 1 and 4 years, parents were asked by their treating healthcare provider for how many hours the orthosis was used. The treating healthcare provider then registered the reported number of hours according to fixed categories; >10 h/night, 6–10 h/night, < 6 h/night in the register. In case of change of orthosis type, the fixed categories possible to report reasons for changing orthosis type were: “Abrasions”, “Parental request”, “Sleep disturbances”, or “Other”. Providing a reason for changing orthosis type was not mandatory, therefore this data was not included in the present study.

### The PBS-score

The PBS-score was published in 2019 as a tool to grade the severity of clubfoot deformity in ambulatory children [26]. In 2022, a recommended core outcome set for evaluating children with clubfoot was published in which the PBS-score was included [27]. The score ranges from 7 (no foot deformity) to 18 (severe foot deformity), and is based on evaluations in standing (hind foot varus, fixed supination), walking (swing phase supination, early heel rise), passive subtalar abduction, active and passive ankle dorsiflexion [26]. The PBS-score is registered in SPOQ when the child is 4 years of age.

### Statistical analyses

Statistical analyses were performed using the Statistical Package for Social Sciences, version 25 (SPSS Inc., Chicago, IL, USA) with *p* < 0.05 determining statistical significance. Demographics and disease characteristics were described using median, minimum, maximum, frequency, and/or percent.

A chi^2^ test was used to; estimate differences in the proportion of boys/girls, uni-/bilateral involvement, the number of feet with atypical signs between children with isolated clubfoot and those with non-isolated clubfoot, the proportion of parents reporting orthotic use > 10 h/day at 1 and 4 years of age, and the proportion of children changing orthosis type before or at/after 4 years of age. The Mann-Whitney U test was used to evaluate differences in the Pirani score at diagnosis between children with isolated and non-isolated clubfoot, and between children with isolated clubfoot ending orthotic treatment before or at/after 4 years of age. To compare differences in the PBS-score at 4 years of age between children ending orthotic treatment before 4 years of age and children continuing orthotic treatment to 4 years of age or longer, the Mann-Whitney U test was used. To account for the effect of bilateral disease when evaluating the treatment, only one foot from children with bilateral involvement was included. The right or left foot was included for every second child based on the inclusion number in the register, allowing an even spread across centers and years.

## Results

### Participants

Of the 387 children registered in SPOQ between 2015 and 2017, clubfoot status at 4 years of age was reported in a total of 321 children. Of the 321 included children, 288 (90%) had isolated clubfoot. Thirty-three children (10%) had a non-isolated clubfoot. Details are shown in Table 1. An overview of included data presented on group level are shown in Appendix 1.

### Missing data

Registration at 4 years of age was missing for 66 children out of the 387 children. Thus, they did not meet the inclusion criteria and were not included in the present study. Out of these 66 children, 56% had bilateral involvement, and 71% were boys, both comparable numbers with the included children with a 4-year follow-up. 23% had non-isolated clubfoot which was a significantly higher proportion as compared to the included children with a registered 4-year follow-up (*p* = 0.05). Hence, children without registration in SPOQ at 4 years of age had a non-isolated clubfoot to a greater extent. Out of the 321 included children, twenty-seven children (8%) did not have a 1-year registration, and for these children data on parent-reported orthotic use at the 1-year follow-up is missing. In 2015, when the SPOQ-registrations started, all variables were not mandatory. Therefore, a few cases of missing data were noted, and these are reported in specific in relation to each outcome and in Appendix 1.

**Table 1:** Demographic description of the included children with clubfoot

	Total population (*n* = 321)	Isolated clubfoot (*n* = 288)	Non-isolated clubfoot (*n* = 33)	Differences between children with isolated and non-isolated clubfoot
Boys (n (%))	229 (71)	207 (72)	22 (67)	0.67^1^
Bilateral (n (%))	156 (49)	144 (50)	21 (64)	0.14^1^
Atypical clubfoot (n (%))	24 (7.5)	16 (6)	8 (24)	**< 0.001** ^**1**^
Pirani score at diagnosis (median (min, max))	4.5 (1, 6)	4.5 (1, 6)	5.5 (1.5, 6)	**< 0.004** ^**2**^
Non-isolated clubfoot:				
Arthrogryposis multiplex congenita (n (%))	7 (2)	N.A	7 (21)	
Spina bifida (n (%))	3 (1)	N.A	3 (9)	
Congenital malformation syndromes* (n (%))	5 (2)	N.A	5 (15)	
Neurological diseases (n (%))	5 (2)	N.A	5 (15)	
Other, not specified (n (%))	13 (4)	N.A	13 (39)	

N, number of clubfeet/children; N.A, Not Applicable; ^1^, Chi^2^ test; ^2^, Mann-Whitney U test; *, predominantly involving limbs

### Prescribed type of orthosis

#### Isolated clubfoot

Of the 288 children with isolated clubfoot, 275 children (95%) were prescribed an FAO, seven children (2%) a KAFO, and two children (1%) an AFO. Two children (1%) were prescribed other, non-specified orthosis, and two children (1%) were not prescribed any orthosis.

#### Non-isolated clubfoot

Of the 33 children with non-isolated clubfoot, 25 children (76%) were prescribed an FAO, four children (12%) a KAFO, and two children (6%) an AFO. Two children (6%) were prescribed other, non-specified orthosis.

### Prescribed orthotic use

#### Isolated clubfoot

From the start, 266 children (92%) with isolated clubfoot were prescribed to use the orthosis 23 h/day for the first three months, 16 children (6%) were prescribed 18 h/day, and two children (1%) 10–14 h/day. The information was missing for four children (1%).

#### Non-isolated clubfoot

From the start, 30 children (91%) with isolated clubfoot were prescribed to use the orthosis 23 h/day for the first three months, and two children (6%) were prescribed 18 h/day. No child was prescribed 10–14 h/day. The information was missing for one child (3%).

### Parent-reported orthotic use

**Fig. 2:**
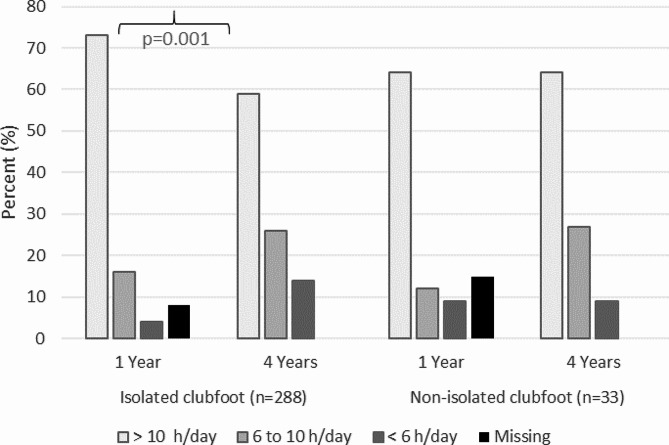
Parent-reported orthotic use at 1 and 4 years of age

The number of parents reporting time of orthotic use > 10 h/day decreased significantly between 1 and 4 years of age for children with isolated clubfoot *p* = 0.001), while the reported time of > 10 h/day remained stable over time for children with non-isolated clubfoot (Fig. 2).

#### Isolated clubfoot

At 1 year of age, 210 children (73%) with isolated clubfoot used their orthosis > 10 h/night, 45 children (16%) used their orthosis between 6 and 10 h/night, and 11 children (4%) used their orthosis < 6 h/night. The information was missing for 22 children (7%). At 4 years of age, or when ending orthotic treatment, 171 children (60%) with isolated clubfoot used the orthosis > 10 h/night, 76 children (26%) used their orthosis between 6 and 10 h/night, and 41 children (14%) used their orthosis < 6 h/night (Fig. 2).

#### Non-isolated clubfoot

At 1 year of age, 21 children (64%) with non-isolated clubfoot used their orthosis > 10 h/night, four children (12%) used the orthosis between 6 and 10 h/night, and three children (9%) used their orthosis < 6 h/night. The information was missing for five children (15%). At 4 years of age, or when ending orthotic treatment, 21 children (64%) with non-isolated clubfoot used their orthosis > 10 h/night, 9 children (27%) used the orthosis between 6 and 10 h/night, and 3 children (9%) used the orthosis < 6 h/night (Fig. 2).

### Change of orthosis type

#### Isolated clubfoot

Of the 288 children with isolated clubfoot, 100 children (35%) changed orthosis type, e.g. from FAO to KAFO or AFO, before 4 years of age. Of these, 51 children changed orthosis-type once, and 49 children changed twice or more times.

#### Non-isolated clubfoot

Of the 33 children with non-isolated clubfoot, 21 children (64%) changed orthosis type before 4 years of age. Of these, 8 children changed orthosis type once, and 13 children changed twice or more times.

### Duration of orthotic treatment

**Fig. 3:**
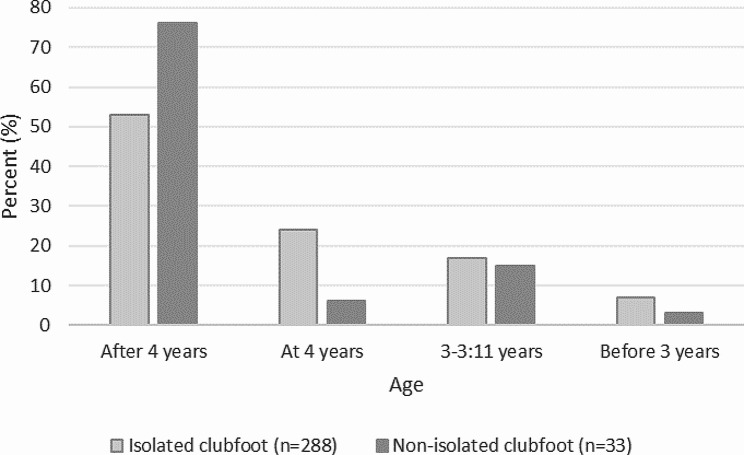
Parent-reported end of orthotic treatment

Of the 228 children with isolated clubfoot, 220 (76%) continued with orthotic treatment until 4 years of age or longer (Fig. 3). Of the 33 children with non-isolated clubfoot, 28 (84%) continued with orthotic treatment until 4 years of age or longer (Fig. 3).

### Characteristics of children with isolated clubfoot changing orthosis type and ending orthotic treatment before age 4

Children with isolated clubfoot ending orthotic treatment before 4 years of age (*n* = 63, median: 4.5, IQR: 3.0, 5.0) had significantly lower Pirani score at birth compared to children ending orthosis treatment at/after 4 years of age (*n* = 219, median 4.5, IQR: 4.0, 5.5) (*p* = 0.01). While not statistically different, children with isolated unilateral clubfoot ended orthosis treatment more often before 4 years of age compared to children with isolated bilateral clubfoot (28% vs. 19%, *p* = 0.052).

Children ending orthotic treatment before 4 years of age changed orthosis type statistically significantly more often (*n* = 31, 46%) compared to children ending orthosis treatment at/after 4 years of age (*n* = 69, 31%) (*p* = 0.031). Children with unilateral isolated clubfoot changed orthosis type more often as compared to children with bilateral clubfoot (unilateral: 64 children (44%), vs. bilateral: 36 children (25%), *p* < 0.001).

No difference was found in PBS-score at 4 years of age between children ending orthotic treatment before 4 years of age (*n* = 68, median: 7, IQR: 7.0, 9.0) or at/after age 4 (*n* = 219, median 7, IQR: 7.0, 8.0) (*p* = 0.08).

## Discussion

This study aimed to examine the duration, course, and outcome of orthotic treatment in children with clubfoot deformity. To this end, data from a national Swedish register of children born 2015–2017 were extracted and analyzed. Results of this study demonstrate that the vast majority of children are prescribed and treated with an FAO during the maintenance phase. This aligns well with the results of a recent survey conducted among clubfoot practitioners within the Pediatric Orthopedic Society of North America (POSNA) that aimed to identify current treatment practices [28]. The maintenance phase is often highlighted as the most important treatment phase, where longer duration of treatment period results in less recurrences [29, 30]. The FAO is the first-choice orthosis type according to the Ponseti method, and it has shown superior results, in addition to most often being well tolerated by children and families [6, 8]. Ending orthotic treatment prematurely is a well-recognized problem associated with an increased risk of recurrence [31]. In the studied cohort, only two children, both with an isolated clubfoot, out of 321 were *not* prescribed any orthosis when transitioning from initial correction to maintenance phase.

In clinical practice, families of children with unilateral clubfoot more often question the FAO as it is applied on both feet. The utility of a unilateral orthosis has been debated, and while some studies have found a higher recurrence rate among children using unilateral orthoses, other studies report results that are comparable to results of using an FAO [32, 33]. Most clinicians agree that using an(y) orthosis is better than not using one at all, and to date, most scientific results are in favor of the FAO [31, 34, 35].

The results showed that the proportion of children *changing orthosis type* during the maintenance phase was significantly greater among children ending orthotic treatment early. While not statistically significant, data further indicate that children with isolated unilateral clubfoot ended orthotic treatment prematurely to a higher extent than children with bilateral involvement. This may be interpreted that a request of changing orthosis type should be viewed as a warning signal for insufficient compliance and ending the maintenance phase prematurely. Thus, if change of orthosis type is discussed, additional support from health care providers should be offered. Families must be supported, encouraged, and educated that compliance to orthotic treatment is the most important factor for preventing recurrence of foot deformity [29, 31].

In the studied cohort, 76% of the children with isolated clubfoot and 84% of the children with non-isolated clubfoot continued with orthotic treatment until the age of 4 years or longer, indicating good compliance with the Ponseti method on a national level. However, 60% of the parents reported an orthotic use time at 4 years of > 10 h/day, indicating a decrease in orthotic use time between 1 and 4 years of age. The present study aimed to analyze characteristics among children ending orthosis treatment before 4 years of age. The 4-years evaluation in SPOQ correspond to when most clinics end orthotic treatment according to their local protocols, as well as to the recommendation by Ponseti international by the time that SPOQ was founded (in 2015) [6, 8]. In recent years it has become increasingly common to extend orthotic treatment until 5 years of age with the goal to prevent recurrence [36]. Our results showed that children with isolated clubfoot ending orthotic treatment before 4 years of age had significantly lower Pirani scores at birth. Future research is required to identify the importance of this result. It remains unknown whether the consequences of ending orthotic treatment prematurely are decisive or not for children with a low Pirani score at birth. No difference was found in PBS-score at 4 years of age between children ending orthotic treatment before 4 years of age or at/after age 4. Future studies should focus on evaluating the long-term effect of duration of orthotic treatment on functional outcomes. Furthermore, longer-term follow-up studies are required to accurately predict the ultimate risk of recurrent foot deformity [16]. Thomas et al. conclude in their systematic review of recurrence rates that patients and their care-givers should be aware of the possibility of recurrence during middle and late childhood, i.e. years after ending orthotic treatment [16]. Furthermore, future data from SPOQ will expand current knowledge of late recurrence and the relation to orthotic treatment, since children will be followed until 18 years of age.

This study holds several limitations that need to be acknowledged. In the register, it was not mandatory to provide any reasons for early drop-out of the orthotic treatment protocol, nor was it mandatory to provide the primary reason for changing orthosis type. Thus, no conclusions can be drawn as of *why* orthotic treatment ended before the age of 4, or the primary reasons for changing orthosis type. The current data set did not include information on recurrence. Thus, no analyses regarding recurrence in relation to orthotic treatment could be performed. To the best of our knowledge, all reporting clinics encourage orthotic use to at least 4 years of age, and encourage orthotic use of 12 h/night. However, the maximum option for parent-reported orthotic use-time in SPOQ was set to 10 h/night. This time-option was set to apply also for 4-year-old children that may sleep less than 12 h/night [37], but that are still compliant with the treatment regime. Finally, no additional data was collected and registered in SPOQ between the 1-year and 4-year registrations. A recent study reported decreased orthotic use time over a three-month period in children aged 2 years. By the second year of orthotic treatment, nearly half of the patients wore their orthoses 8 h or less [38]. With that in mind, results of the present study cannot inform when in time the decrease of orthotic use occurred.

This is the first study evaluating change of orthosis type and how this relates to ending orthotic treatment prematurely. While children with clubfoot in Sweden are treated according to the Ponseti method, there is no national consensus on when to end the orthotic treatment. The present study includes a national cohort with prospectively and longitudinally collected data which is considered as a strength. The included data points are well-defined and described on the register website, and there are regular user-meetings for clinicians and administrative staff involved in the registration process [11]. Furthermore, the directors of the clubfoot register are continuously in contact with pediatric orthopedic centers when needed to assist with interpretation of scales, and to encourage registration.

## Conclusion

Results of this study demonstrate that the vast majority of children with clubfoot in Sweden are prescribed and treated with an FAO during the maintenance phase. The proportion of children *changing orthosis type* during the maintenance phase was significantly greater and the Pirani score at diagnosis was significantly lower among children ending orthotic treatment before the age of 4 years. Thus, requests to change orthosis type may indicate insufficient compliance. Longer term follow-up studies are warranted to fully understand how to optimize, and individualize, orthotic treatment with respect to foot involvement and severity of deformity.

### Electronic supplementary material

Below is the link to the electronic supplementary material.

Supplementary Material 1

## Data Availability

Availability of data and material: Legal restrictions prevent this data from being publicly (Public Access and Secrecy, chapter 21, paragraph 7 and chapter 25, paragraph 1 (https://www.riksdagen.se/sv/dokument-lagar/dokument/svensk-forfattningssamling/offentlighets--och-sekretesslag-2009400_sfs-2009-400). The data included in this research project was anonymized but due to small dataset, especially when divided into healthcare regions, we cannot guarantee participant privacy. For permission to access data from SPOQ, the reader is referred to the Center of Registers, Västra Götaland (URL: http://registercentrum.se/).

## Abbreviations

AFO: Ankle-Foot-Orthosis
FAO: Foot Abduction Orthosis
IQR: Interquartile Range
KAFO: Knee-Ankle-Foot-Orthosis
PBS-score: Pirani Böhm Sinclair-score
SPOQ: Swedish Pediatric Orthopedic Quality Register

## References

[1] Wang H, Barisic I, Loane M, Addor MC, Bailey LM, Gatt M (2019). Congenital clubfoot in Europe: a population-based study. Am J Med Genet A.

[2] Moon DK, Gurnett CA, Aferol H, Siegel MJ, Commean PK, Dobbs MB (2014). Soft-tissue abnormalities associated with treatment-resistant and treatment-responsive clubfoot: findings of MRI analysis. J Bone Joint Surg Am.

[3] Dobbs MB, Gurnett CA (2012). Genetics of clubfoot. J Pediatr Orthop B.

[4] Esbjornsson AC, Johansson A, Andriesse H, Wallander H (2021). Epidemiology of clubfoot in Sweden from 2016 to 2019: a national register study. PLoS ONE.

[5] Abraham J, Wall JC, Diab M, Beaver C (2021). Ponseti Casting vs. Soft tissue release for the initial treatment of non-idiopathic clubfoot. Front Surg.

[6] Ponseti IV (1996). Congenital clubfoot. Fundamentals of treatment.

[7] Agarwal A, Shanker M (2020). Correlation of scores with number of Ponseti casts required for clubfoot correction in the older child. J Clin Orthop Trauma.

[8] Rieger MA, Dobbs MB, Clubfoot (2022). Clin Podiatr Med Surg.

[9] Association PI. Clinical Practice Guidelines for the Management of Clubfoot Deformity Using the Ponseti Method http://www.ponseti.info/home.html2015. [Accessed 7 Jan 2024].

[10] Ponseti IV, Becker JR (1966). Congenital metatarsus adductus: the results of treatment. J Bone Joint Surg Am.

[11] spoq.registercentrum.se [Internet]. SPOQ, Svenskt Pediatriskt Ortopediskt Qualitetsregister; c2023 [cited 2023 May 11]. https://spoq.registercentrum.se/. [Accessed 11 May 2023].

[12] Ponseti IV (1994). The treatment of congenital clubfoot. J Orthop Sports Phys Ther.

[13] Zionts LE, Sangiorgio SN, Ebramzadeh E, Morcuende JA (2012). The current management of idiopathic clubfoot revisited: results of a survey of the POSNA membership. J Pediatr Orthop.

[14] Zionts LE, Zhao G, Hitchcock K, Maewal J, Ebramzadeh E (2010). Has the rate of extensive surgery to treat idiopathic clubfoot declined in the United States?. J Bone Joint Surg Am.

[15] Gelfer Y, Wientroub S, Hughes K, Fontalis A, Eastwood DM (2019). Congenital talipes equinovarus: a systematic review of relapse as a primary outcome of the Ponseti method. Bone Joint J.

[16] Thomas HM, Sangiorgio SN, Ebramzadeh E, Zionts LE (2019). Relapse Rates in patients with clubfoot treated using the Ponseti Method increase with time: a systematic review. JBJS Rev.

[17] Agarwal A, Rastogi A, Rastogi P (2021). Relapses in clubfoot treated with Ponseti technique and standard bracing protocol- a systematic analysis. J Clin Orthop Trauma.

[18] Rastogi A, Agarwal A (2021). Long-term outcomes of the Ponseti method for treatment of clubfoot: a systematic review. Int Orthop.

[19] Zionts LE, Ebramzadeh E, Sangiorgio SN (2021). Objective analysis of intermediate-term outcome of the Ponseti technique: a review of the experience from Los Angeles. Ann Transl Med.

[20] Hosseinzadeh P, Kelly DM, Zionts LE (2017). Management of the relapsed clubfoot following treatment using the Ponseti Method. J Am Acad Orthop Surg.

[21] Ponseti IV (2002). Relapsing clubfoot: causes, prevention, and treatment. Iowa Orthop J.

[22] Stouten JH, Besselaar AT, Van Der Steen MCM (2018). Identification and treatment of residual and relapsed idiopathic clubfoot in 88 children. Acta Orthop.

[23] Shaheen S, Jaiballa H, Pirani S (2012). Interobserver reliability in Pirani clubfoot severity scoring between a paediatric orthopaedic surgeon and a physiotherapy assistant. J Pediatr Orthop B.

[24] Jain S, Ajmera A, Solanki M, Verma A (2017). Interobserver variability in Pirani clubfoot severity scoring system between the orthopedic surgeons. Indian J Orthop.

[25] Bettuzzi C, Abati CN, Salvatori G, Zanardi A, Lampasi M (2019). Interobserver reliability of Dimeglio and Pirani score and their subcomponents in the evaluation of idiopathic clubfoot in a clinical setting: a need for improved scoring systems. J Child Orthop.

[26] Bohm S, Sinclair MF (2019). The PBS score - a clinical assessment tool for the ambulatory and recurrent clubfoot. J Child Orthop.

[27] Gelfer Y, Hughes KP, Fontalis A, Wientroub S, Eastwood DM (2020). A systematic review of reported outcomes following Ponseti correction of idiopathic club foot. Bone Jt Open.

[28] Sax OC, Hlukha LP, Herzenberg JE, McClure PK. Current clubfoot practices: POSNA Membership Survey. Child (Basel). 2023;10(3). 10.3390/children10030439 PMC10047051 36979996

[29] Cady R, Hennessey TA, Schwend RM. Diagnosis and treatment of idiopathic congenital clubfoot. Pediatrics. 2022;149(2). 10.1542/peds.2021-055555 PMC9645716 35104362

[30] Garg S, Porter K (2009). Improved bracing compliance in children with clubfeet using a dynamic orthosis. J Child Orthop.

[31] Desai L, Oprescu F, DiMeo A, Morcuende JA (2010). Bracing in the treatment of children with clubfoot: past, present, and future. Iowa Orthop J.

[32] Berger N, Lewens D, Salzmann M, Hapfelmeier A, Doderlein L, Prodinger PM (2018). Is unilateral lower leg orthosis with a circular foot unit in the treatment of idiopathic clubfeet a reasonable bracing alternative in the Ponseti method? Five-year results of a supraregional paediatric-orthopaedic centre. BMC Musculoskelet Disord.

[33] Farrar EJ, Lo M, Groothoff L, Cunningham J, Theuri J (2022). Two-year retrospective cohort results on use of a dynamic unilateral brace for treatment of clubfoot: can compliance and prevention of recurrence both be achieved?. J Rehabil Assist Technol Eng.

[34] George HL, Unnikrishnan PN, Garg NK, Sampath J, Bruce CE (2011). Unilateral foot abduction orthosis: is it a substitute for Denis Browne boots following Ponseti technique?. J Pediatr Orthop B.

[35] Janicki JA, Wright JG, Weir S, Narayanan UG (2011). A comparison of ankle foot orthoses with foot abduction orthoses to prevent recurrence following correction of idiopathic clubfoot by the Ponseti method. J Bone Joint Surg Br.

[36] Alves C (2019). Bracing in clubfoot: do we know enough?. J Child Orthop.

[37] Paruthi S, Brooks LJ, D’Ambrosio C, Hall WA, Kotagal S, Lloyd RM (2016). Recommended amount of Sleep for Pediatric populations: a Consensus Statement of the American Academy of Sleep Medicine. J Clin Sleep Med.

[38] Richards BS, Faulks S, Felton K, Karacz CM (2020). Objective measurement of Brace wear in successfully Ponseti-treated clubfeet: pattern of decreasing Use in the first 2 years. J Am Acad Orthop Surg.

