# Supplementary material for: Duration and outcome of orthotic treatment in children with clubfoot – a four-year follow-up national register study of Swedish children born between 2015 and 2017
Source: BMC Musculoskelet Disord. 2024 May 31;25:425. doi: 10.1186/s12891-024-07544-5 (PMC11143618; doi:10.1186/s12891-024-07544-5)
Supplement: Supplementary file 1 — Supplementary Material 1 [file 12891_2024_7544_MOESM1_ESM.docx]

**Appendix 1. Overview of included data presented on group level.**

|  | **Total population**  (n=321) | **Isolated**  **clubfoot**  (n=288) | **Non-isolated clubfoot**  (n=33) |
| --- | --- | --- | --- |
| Boys, n (%) | 229 (71) | 207 (72) | 22 (67) |
| Bilateral, n (%) | 156 (49) | 144 (50) | 21 (64) |
| Atypical clubfoot, n (%) | 24 (7.5) | 16 (6) | 8 (24) |
| Pirani score at diagnosis, median (min, max) | 4.5 (1–6) | 4.5 (1–6) | 5.5 (1.5–6) |
|  |  |  |  |
| **Non-isolated clubfoot** | **n (%)** | | |
| Arthrogryposis multiplex congenita | 7 (2) | N.A | 7 (21) |
| Spina bifida | 3 (1) | N.A | 3 (9) |
| Congenital malformation syndromes* | 5 (2) | N.A | 5 (15) |
| Neurological diseases | 5 (2) | N.A | 5 (15) |
| Other, not specified | 13 (4) | N.A | 13 (39) |
|  |  |  |  |
| **Prescribed type of orthosis** |  | **n (%)** |  |
| Foot abduction orthosis (FAO) | 300 (93) | 275 (96) | 25 (76) |
| Knee-ankle-foot-orthosis (KAFO) | 11 (3) | 7 (2) | 4 (12) |
| Non-specified orthosis | 4 (1) | 2 (1) | 2 (6) |
| No orthosis | 4 (1) | 2 (1) | 2 (6) |
| Missing data | 2 (1) | - | - |
|  |  |  |  |
| **Prescribed orthotic use from the start** |  | **n (%)** |  |
| 23 h/day | 296 (92) | 266 (92) | 30 (91) |
| 18 h/day | 18 (6) | 16 (6) | 2 (6) |
| 10-14 h/day | 2 (1) | 2 (1) | 0 (0) |
| Missing data | 5(1) | 4 (1) | 1 (3) |
|  |  |  |  |
| **Parent-reported orthotic use at 1 year of age** |  | **n (%)** |  |
| >10 h/night | 231 (72) | 210 (73) | 21 (64) |
| 6-10 h/night | 49 (15) | 45 (16) | 4 (12) |
| <6 h/night | 14 (5) | 11 (4) | 3 (9) |
| Missing data | 27 (8) | 22 (7) | 5 (15) |
|  |  |  |  |
| **Parent-reported orthotic use at 4 years of age** |  | **n (%)** |  |
| >10 h/night | 192 (60) | 171 (60) | 21 (64) |
| 6-10 h/night | 85 (26) | 76 (26) | 9 (27) |
| <6 h/night | 44 (14) | 41 (14) | 3 (9) |
|  |  |  |  |
| **Number of children changing orthosis type** |  | **n (%)** |  |
| From FAO to KAFO/AFO | 121 (38) | 100 (35) | 21 (64) |
| Once | 59 (49) | 51 (51) | 8 (38) |
| Two or more times | 62 (51) | 49 (49) | 13 (62) |
|  |  |  |  |
| **Duration of orthotic treatment** |  | **n (%)** |  |
| Continued use until 4 years of age (or longer) | 248 (77) | 220 (76) | 28 (85) |
| End before 4 years of age | 73 (23) | 68 (24) | 5 (15) |
|  |  |  |  |

N, number of clubfeet/children; N.A, Not Applicable; *, predominantly involving limbs.
